# Hepatitis B virus (HBV) status of children born to HIV/HBV co-infected women in a French hospital: a cross-sectional study

**DOI:** 10.7448/IAS.17.4.19632

**Published:** 2014-11-02

**Authors:** Pierre Sellier, Nathalie Schnepf, Rishma Amarsy, Sarah Maylin, Martin Coutellier, Amanda Lopes, Marie-Christine Mazeron, Clara Flateau, Marjolaine Morgand, Nicole Ciraru-Vigneron, Aurore Berthe, Aude Ricbourg, Anne-Marie Dolores-Moreno, Guy Simoneau, John Evans, Safia Souak, Sophie Matheron, Stephane Mouly, Jean-Louis Benifla, François Simon, Jean-François Bergmann

**Affiliations:** 1 Internal Medicine, Hopital Lariboisière 75010, Paris, France; 2 Hopital Saint-Louis 75010, Virologie, Paris, France; 3 Hopital Lariboisière 75010, Virologie, Paris, France; 4 Hopital Bichat-Claude Bernard, Service des Maladies Infectieuses et Tropicales, Paris, France; 5 Hopital Lariboisière 75010, Gynecologie-Obstetrique, Paris, France; 6 Hopital Lariboisière 75010, Pharmacie, Paris, France

## Abstract

**Introduction:**

Human Immunodeficiency Virus (HIV) Mother-To-Child-Transmission (MTCT) and prevention by combined antiretroviral therapy (cART) have been extensively studied. Hepatitis B Virus (HBV) MTCT from HIV/HBV co-infected women and prevention by antiretroviral therapy with dual activity have been poorly studied. The aim of the study was to assess HBV MTCT from HIV/HBV co-infected women in a developed country with a large access to cART.

**Materials and Methods:**

HIV/HBV co-infected pregnant women attending the Obstetrics Department from 1st January 2000 to 1st January 2012 could be included in the study (NCT02044068). Antiretroviral therapy during pregnancy, injection of immunoglobulin and/or vaccine to newborns was retrospectively recorded. We assessed HBV status of children at least as old as two years.

**Results:**

Forty nine (9.2%) from 530 HIV-infected women followed in the hospital were HIV/HBV co-infected. 34 (69.4%) had given birth to 57 children in the hospital. 13 of these women (22 children) were lost-to-follow-up, 21 women (35 children) could be studied. Twenty six children (74.3%) had HBs Ab at a protective level, 22 of them had received immunoglobulin at birth; 24 had received a complete vaccine schedule during the first six months of life (with immunoglobulin in 21 cases). The women had been given lamivudine or tenofovir/emtricitabine during eight and nine pregnancies respectively. Eight children (22.8%) were tested negative for HBs Ag, HBs Ab and HBc Ab: 4 (11.4%) had received immunoglobulin and a complete vaccine schedule; in two children, immunoglobulin was uncertain; in one child, the vaccine schedule was incomplete; in the last one, data about immunoglobulin and the vaccine schedule were lacking. The women had been given lamivudine or tenofovir/emtricitabine during five and two pregnancies respectively. One child had HBc Ab and HBs Ab, immunoglobulin was uncertain and the vaccine schedule was incomplete. The woman had been given lamivudine during the last trimester.

**Conclusions:**

Three quarters of the children were protected. HBs Ab were negative in more than a tenth of the children who had received immunoglobulin and a complete vaccine schedule, questioning on long-term protection and underlining the need of control.

